# Ray-tracing analytical absorption correction for X-ray crystallography based on tomographic reconstructions

**DOI:** 10.1107/S1600576724002243

**Published:** 2024-04-15

**Authors:** Yishun Lu, Ramona Duman, James Beilsten-Edmands, Graeme Winter, Mark Basham, Gwyndaf Evans, Jos J. A. G. Kamps, Allen M. Orville, Hok-Sau Kwong, Konstantinos Beis, Wesley Armour, Armin Wagner

**Affiliations:** a Oxford e-Research Centre, Department of Engineering Science, University of Oxford, 7 Keble Road, Oxford OX1 3QG, United Kingdom; b Diamond Light Source, Harwell Science & Innovation Campus, Didcot OX11 0DE, United Kingdom; c Rosalind Franklin Institute, Harwell Science & Innovation Campus, Didcot OX11 0QX, United Kingdom; d Rutherford Appleton Laboratory, Research Complex at Harwell, Didcot OX11 0FA, United Kingdom; e Department of Life Sciences, Imperial College London, Exhibition Road, London SW7 2AZ, United Kingdom; DESY, Hamburg, Germany

**Keywords:** absorption correction, ray tracing, long-wavelength crystallography, X-ray tomography

## Abstract

Processing of single-crystal X-ray diffraction data from area detectors can be separated into two steps. First, raw intensities are obtained by integration of the diffraction images, and then data correction and reduction are performed to determine structure-factor amplitudes and their uncertainties. The second step considers the diffraction geometry, sample illumination, decay, absorption and other effects. While absorption is only a minor effect in standard macromolecular crystallography (MX), it can become the largest source of uncertainty for experiments performed at long wavelengths. Current software packages for MX typically employ empirical models to correct for the effects of absorption, with the corrections determined through the procedure of minimizing the differences in intensities between symmetry-equivalent reflections; these models are well suited to capturing smoothly varying experimental effects. However, for very long wavelengths, empirical methods become an unreliable approach to model strong absorption effects with high fidelity. This problem is particularly acute when data multiplicity is low. This paper presents an analytical absorption correction strategy (implemented in new software *AnACor*) based on a volumetric model of the sample derived from X-ray tomography. Individual path lengths through the different sample materials for all reflections are determined by a ray-tracing method. Several approaches for absorption corrections (spherical harmonics correction, analytical absorption correction and a combination of the two) are compared for two samples, the membrane protein OmpK36 GD, measured at a wavelength of λ = 3.54 Å, and chlorite dismutase, measured at λ = 4.13 Å. Data set statistics, the peak heights in the anomalous difference Fourier maps and the success of experimental phasing are used to compare the results from the different absorption correction approaches. The strategies using the new analytical absorption correction are shown to be superior to the standard spherical harmonics corrections. While the improvements are modest in the 3.54 Å data, the analytical absorption correction outperforms spherical harmonics in the longer-wavelength data (λ = 4.13 Å), which is also reflected in the reduced amount of data being required for successful experimental phasing.

## Abstract 2

A tomography-based ray-tracing approach for analytical absorption corrections for X-ray crystallography is presented. Several examples from long-wavelength macromolecular crystallography experiments demonstrate the efficacy of this new method.

## 1. Introduction

In X-ray crystallography, intensities of reflections are proportional to the square of their structure-factor amplitudes ( 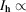 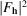 ). Several factors need to be considered when calculating structure-factor amplitudes from measured intensities, such as Lorentz, polarization, sample illumination, decay and absorption corrections (Monaco & Artioli, 2002 ▸). Away from absorption edges, sample absorption is approximately proportional to the cube of the wavelength (Arndt, 1984 ▸). It depends on the chemical composition, density, and shape and size of the sample which includes the crystal, as well as the surrounding materials like sample mount, mother liquor, or oils and glues used to mount the crystals. High-quality structure determination relies on accurate structure-factor amplitudes. Hence, correcting the measured intensities by calculating absorption correction factors is critical. For a crystal which is not surrounded by mother liquor or mounted in a loop, the Bragg intensities after absorption correction are given by 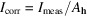 , and the absorption correction factor 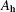 for the reflection **h** in a crystallography experiment is given by 

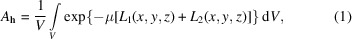

where *L*_1_(*x*, *y*, *z*) and *L*_2_(*x*, *y*, *z*) (hereafter referred to as *L*_1_ and *L*_2_) are the incident and diffracted X-ray path lengths for each crystal element d*V*, and μ is the absorption coefficient of the crystal (Albrecht, 1939 ▸). Since the resulting volumetric integral calculation is intractable for irregularly shaped crystals, absorption correction for multi-faced crystals has been performed by numerical methods (Busing & Levy, 1957 ▸; DeTitta, 1985 ▸). As an alternative approach, the crystal can be partitioned into fundamental tetrahedra to calculate the integral over all the tetrahedra (Howells, 1950 ▸; de Meulenaer & Tompa, 1965 ▸; Clark & Reid, 1995 ▸). Both analytical and numerical absorption corrections require an accurate description of the shape and dimensions of the crystal. One solution from the *APEX3* software (Bruker, 2012 ▸) is to determine and index all the crystal faces visually and perform an analytical absorption correction. However, this is difficult when the shape of the crystal is not a regular polyhedron. In addition, the presence of other materials surrounding the crystal, such as mother liquor and sample mount, adds further complication: these materials with different absorption coefficients only contribute to the absorption effect, not to the diffraction. Semi-empirical methods (North *et al.*, 1968 ▸; Kopfmann & Huber, 1968 ▸) based on intensity measurements and assumptions on the incident and diffracted beams do not rely on knowledge of the sample shape. However, they require multi-axis goniometers, and the additional data needed for the azimuthal scans can contribute significantly to radiation damage on modern synchrotron light sources. Empirical methods which are independent of the sample geometry were developed either based on Fourier series of the incident and diffracted beams (Katayama *et al.*, 1972 ▸; Walker & Stuart, 1983 ▸) or by using spherical harmonics (Blessing, 1995 ▸) to minimize the residual between the intensities for symmetry-related reflections. With the introduction of large area detectors, these numerical methods to obtain an empirical correction for absorption have become popular. Spherical harmonics are now the basis for absorption correction in most data reduction software packages for macromolecular crystallography (MX), such as *AIMLESS* (Evans & Murshudov, 2013 ▸), *hkl3000* (Minor *et al.*, 2006 ▸), *SADABS* (Sheldrick, 1996 ▸) and *DIALS* (Winter *et al.*, 2018 ▸; Beilsten-Edmands *et al.*, 2020 ▸), while *XDS* uses alternative numerical methods without spherical harmonics (Kabsch, 2010 ▸). However, the efficacy of empirical methods depends on having a large number of symmetry-equivalent reflections, which can be difficult to achieve when data multiplicity is low, *e.g.* in the case of radiation-sensitive crystals in low-symmetry space groups.

**Figure 1:**
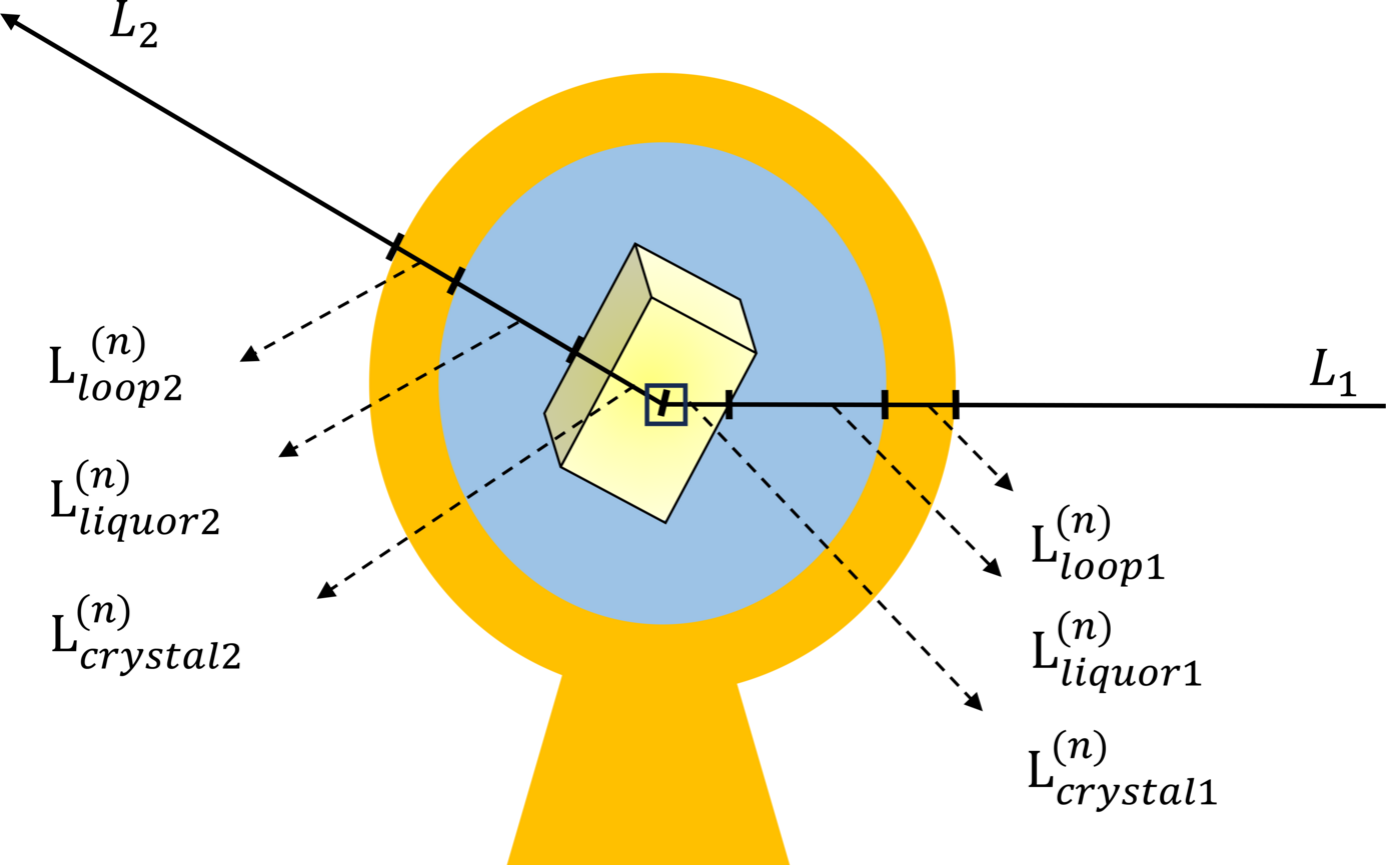
A sketch illustrating the ray-tracing method used to calculate an absorption correction factor for a crystal voxel *n*. 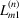 and 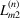 represent the path lengths of the incident and diffracted X-ray beams through the material *m* (loop, liquor and crystal).

As the analytical absorption correction does not depend on refining parameters to minimize differences between structure-factor amplitudes of symmetry-related reflections, its success does not rely on data multiplicity. To analytically calculate absorption correction factors for a sample with irregular shape, its shape and orientation have to be characterized in detail. Previous work using optical microscopy to reconstruct a 3D model of the sample, containing crystal, sample mount and mother liquor, showed that absorption correction was viable and advantageous at lower levels of data multiplicity (Leal *et al.*, 2008 ▸; Strutz, 2011 ▸). An alternative approach to obtain a 3D model of the sample is X-ray tomography, which has been applied to either characterize or visualize crystals (Merrifield *et al.*, 2011 ▸; Warren *et al.*, 2013 ▸). The use of tomographic reconstructions and segmentations as a basis for absorption correction has previously been suggested by Brockhauser *et al.* (2008 ▸). This enables the calculation of X-ray path lengths through the different materials in the sample (crystal, sample mount and mother liquor), as illustrated in Fig. 1 ▸.

While X-ray absorption is not normally considered an issue at standard wavelengths in MX, it is a major limiting factor in long-wavelength crystallography. Beamline I23 at Diamond Light Source, UK (Wagner *et al.*, 2016 ▸), is a unique synchrotron instrument operating in a wavelength range between 1.1 and 5.9 Å, giving access to the absorption edges of several light elements of biological significance, such as calcium, potassium, chlorine, sulfur and phosphorus. The largest anomalous signal for sulfur is expected close to its absorption edge (λ = 5.02 Å). However, the difficulties in correcting for increased sample absorption at very long wavelengths compromise the overall data quality, resulting in reduced measured anomalous signal. Applying standard absorption correction protocols, the optimal wavelength for single-wavelength anomalous diffraction experiments based on sulfur (S-SAD) is found to be λ = 2.75 Å (El Omari *et al.*, 2023 ▸), clearly indicating the need for more sophisticated methods to exploit the full potential of long-wavelength crystallography.

In this paper, we introduce *AnACor*, a computer program that employs a ray-tracing method to estimate the path lengths of the incident and diffracted X-rays through the sample from a tomographic reconstruction, to calculate absorption correction factors for long-wavelength X-ray diffraction data. The effectiveness of *AnACor* is demonstrated for long-wavelength data sets collected at 3.54 Å, on a crystal of the membrane protein OmpK36 GD, and at 4.13 Å, on a crystal of the heme-binding enzyme chlorite dismutase (Cld). OmpK36 GD, referred to as simply ‘OmpK36’, is a 373 amino acid outer membrane porin from *Klebsiella pneumonia* involved in nutrient and antibiotic diffusion in gram negative bacteria (Wong *et al.*, 2019 ▸), while Cld is a heme-*b*-containing homodimeric oxidoreductase from *Cyanothece* sp. PCC7425, consisting of 181 amino acids per monomer. The choice of these two samples for this study was motivated by their crystallization in low-symmetry space groups, posing a challenge for the conventional absorption correction methods used in standard X-ray diffraction scaling programs.

## 2. Methods

### 2.1. Experiment workflow and data preparation

Crystals of OmpK36 were prepared and cryo-protected as previously described with no modification (Wong *et al.*, 2019 ▸). OmpK36 crystallized as rods in space group *C*2, with three monomers present in the asymmetric unit. Large sample-to-sample variations required extensive screening of crystals. The crystal selected for this study had dimensions of 260 × 30 × 30 µm. Cld crystals were produced using a protocol based on previously reported conditions (Schaffner *et al.*, 2017 ▸) with further details provided in the supporting information, section S2. The crystal used in this study had dimensions of 190 × 150 × 90 µm and indexed in space group *P*1, with two monomers in the asymmetric unit.

All experiments were performed at the long-wavelength MX beamline I23 at Diamond Light Source, UK. The in-vacuum sample environment comprises the cylindrical P12M detector and a multi-axis goniometer to enable collection of complete diffraction data from crystals in low-symmetry space groups even at the longest wavelengths. A tomography camera is integrated into the beamline sample environment, allowing easy transition between the two experimental modes (Kazantsev *et al.*, 2021 ▸). The sample preparation for in-vacuum data collection followed the standard protocol for beamline I23 (Duman *et al.*, 2021 ▸). For the OmpK36 crystal, 3 × 360° of data were collected at a wavelength of λ = 3.54 Å with 0.1 s exposure per 0.1° rotation angle and a beam transmission of 50%, with a top-hat X-ray beam adjusted to 240 × 150 µm. To ensure completeness of the data, two of the three data sets were collected using kappa goniometry, with the kappa axis rotated to −70° and the phi axis positioned at 0° and −120°. Each of the three data sets was measured with a photon flux of 1.36 × 10^11^ photons s^−1^, which resulted in a total absorbed dose of 6.5 MGy per data set, as calculated by *Raddose3D* (Zeldin *et al.*, 2013 ▸). Since the Cld crystal diffracted to higher resolution than the OmpK36 crystal, we chose a low-dose data collection strategy. In total 22 × 360° were collected at a wavelength of λ = 4.13 Å with a 350 × 350 µm top-hat beam, using an exposure of 0.1 s per 0.1°. With a beam transmission of 5%, the measured photon flux of 6.7 × 10^9^ photons s^−1^ yielded an absorbed dose of 0.1 MGy per data set. Two of the 22 data sets were collected with the kappa and phi goniometer axes at 0°, while the rest were recorded at κ = −70° and 20 different phi values, between −120° and 120°. The diffraction data were indexed and integrated with *DIALS* (Winter *et al.*, 2018 ▸), providing a kappa/phi orientation matrix, raw intensities, incident vectors, scattering vectors and goniometer angles.

The diffraction experiment was immediately followed by tomography data collection at the same X-ray wavelength. One 180° tomography data set was collected for each crystal, with the kappa and phi axes set at 0° and a beam size of 700 × 700 µm and 100% transmission, using a propagation distance of 4.9 mm between scintillator and sample. For OmpK36 1800 projections, 30 flat-field images (without sample) and 30 dark images (without X-rays) were collected with an exposure of 0.15 s per 0.1° rotation. The measured flux for this data set was 1.5 × 10^12^ photons s^−1^, resulting in a total absorbed dose of 4.8 MGy. For the Cld crystal, 900 projections, 20 flat-field and 20 dark images were collected with an exposure of 0.28 s per 0.2° rotation and a measured flux of 4.3 × 10^11^ photons s^−1^, yielding a total absorbed dose of 0.8 MGy.

**Figure 2:**
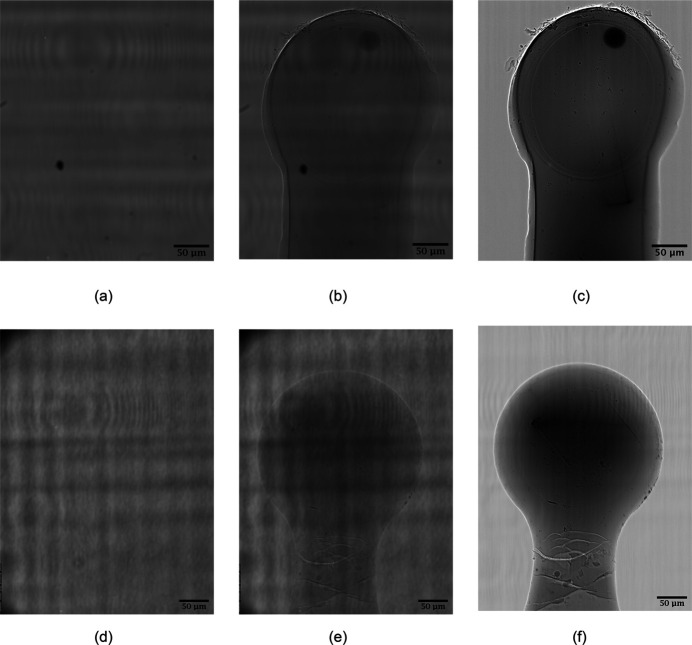
Tomography projection images for background [(*a*) and (*d*)], sample [(*b*) and (*e*)] and flat-field-corrected images [(*c*) and (*f*)] of OmpK36 (top) and Cld samples (bottom).

The tomography data were processed using the *SAVU* pipeline (Kazantsev *et al.*, 2022 ▸), with a processing routine consisting of standard flat-field correction, followed by ring artefact removal (Vo *et al.*, 2018 ▸) and reconstruction. For OmpK36, the reconstruction step was performed by iterative methods via the *ToMoBAR* module in *SAVU* (Kazantsev *et al.*, 2021 ▸), as its edge-enhancing properties gave improved results. For Cld, where the data showed better contrast, the filter-back projection (*TomoPy*) module (Gürsoy *et al.*, 2014 ▸) was used instead. No contrast transfer function correction was applied in the processing. Flat-field images, raw projections and flat-field-corrected projections for both samples are shown in Fig. 2 ▸. For ease of segmentation, reconstruction was performed on cropped data, to eliminate as much of the background as possible and reduce the size of the images. The OmpK36 data were cropped from an initial volume of 1600 × 1200 × 1200 voxels to 1220 × 1001 × 1001 voxels, while the Cld data were reduced to 1310 × 1181 × 1181 voxels. The pixel size in the tomography images, determined from previous beamline calibrations, was 0.3 × 0.3 µm. Manual segmentation was performed with the visualization software *Avizo* (Thermo Fisher), providing a 3D model with every voxel annotated as one of the different sample materials. On the basis of the sample 3D models, the absorption correction factors were calculated and exported to the scaling module in *DIALS* (Beilsten-Edmands *et al.*, 2020 ▸) to further correct the diffraction intensities. Published structures, Protein Data Bank (PDB) ID 6rck (Wong *et al.*, 2019 ▸) for OmpK36, and PDB ID 5mau (Schaffner *et al.*, 2017 ▸) for Cld, were used as starting models for the *Dimple* pipeline (https://ccp4.github.io/dimple/). The ‘- - anode’ option (Thorn & Sheldrick, 2011 ▸) was used to calculate anomalous difference Fourier maps and anomalous peak heights and the option ‘- - free-r-flags’ in the *Refmac* refinement (Murshudov *et al.*, 1997 ▸) step ensured the same *R*_free_ flags for all absorption correction strategies. The *Crank2* phasing pipeline (Skubák & Pannu, 2013 ▸) was used for experimental phasing by single-wavelength anomalous diffraction (SAD) with identical input parameters for the different strategies: the AFRO and PRASA modules were chosen for the F_*A*_ estimation and substructure determination steps, respectively, with the latter step using 4000 trials and resolution cutoffs of 2.7 Å for Cld and 3.4 Å for OmpK36.

### 2.2. Analytical absorption correction

For the calculation of the absorption correction factors, the integral [equation (1)] is calculated over the crystal volume (Angel, 2004 ▸) as the only source of X-ray diffraction. To move from the continuous integral in equation (1) to a discrete equation, we replace crystal elements d*V* by crystal voxels Δ*V* from the tomographic reconstruction (Leal *et al.*, 2008 ▸). This allows substitution of the integral over the volume *V* with a sum over the crystal voxels. Hence, the integral in equation (1) can be rewritten discretely as 

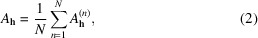

where *N* is the number of crystal voxels in the 3D model exposed to the X-ray beam. The sample in a crystallography experiment typically contains more than one material; therefore, the calculation of the absorption correction factor 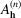 for a crystal voxel can be rewritten as

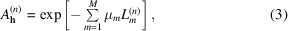

where 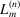 represents the sum of the incident path length 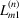 and the diffracted path length 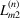 through the material *m* as shown in Fig. 1 ▸.

The final squared structure-factor amplitudes 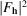 are obtained after combining their absorption correction factors with the overall scale factor, Lorentz and polarization corrections, and other standard correction and scaling techniques.

### 2.3. Absorption coefficients

**Figure 3:**
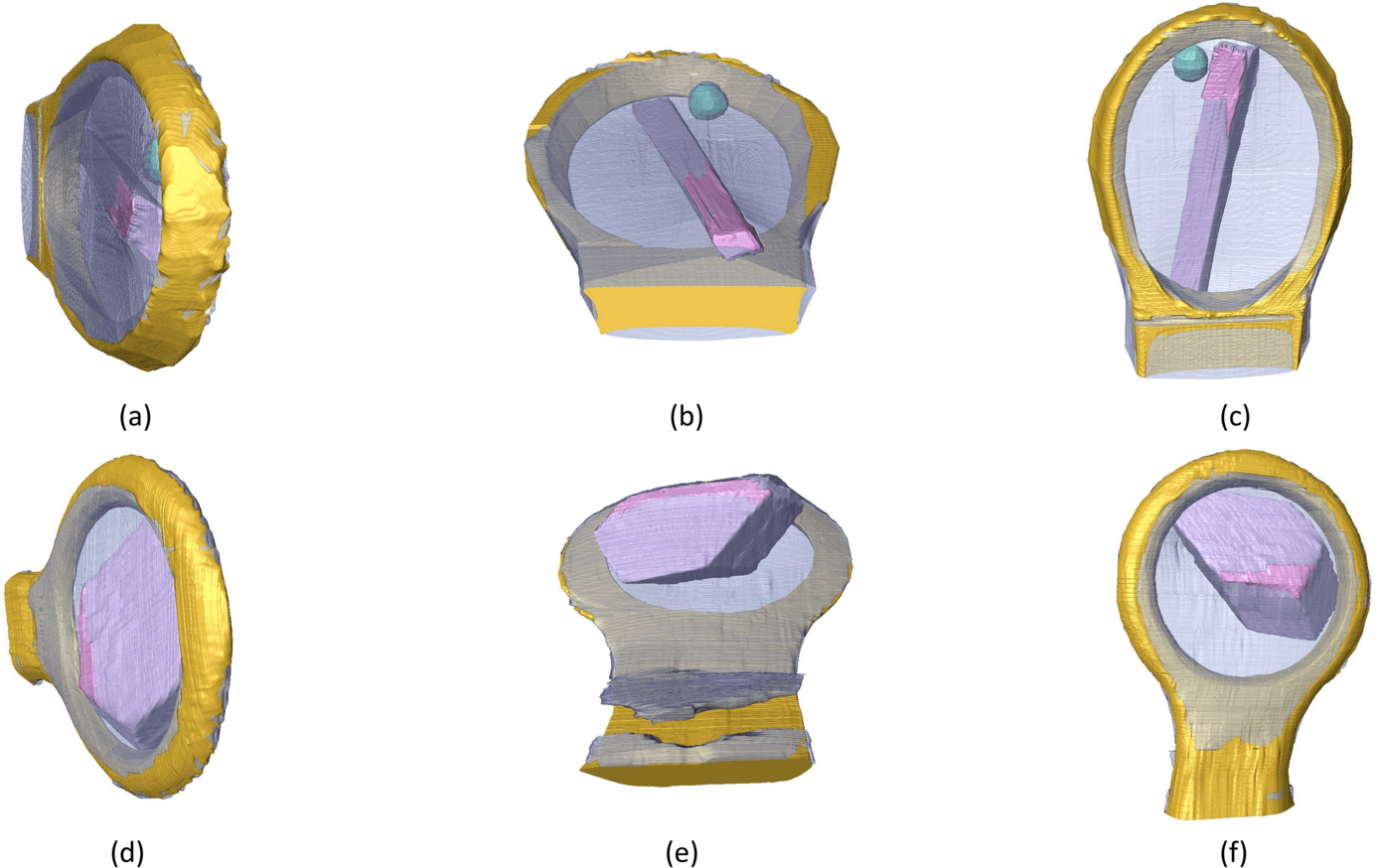
Volume renderings of segmentations of OmpK36 [(*a*)–(*c*)] and Cld [(*d*)–(*f*)]. Transparent blue: mother liquor; gold: loop; pink: crystal; green: protein/detergent aggregate.

Absorption coefficients are determined experimentally using the intensity values in the flat-field-corrected tomograms [Figs. 2 ▸(*c*), 2 ▸(*f*)] as estimates of the ratio between the incident and transmitted intensities. The distances through each material required for the calculation are obtained from the 3D segmentation models. The 3D models of the OmpK36 and Cld samples in different orientations are presented in Fig. 3 ▸. To make sure the transmitted intensities on the tomograms and the path lengths from the segmentation model are aligned, a Python script is used to superpose the 2D projection of the model onto the tomogram. The areas of the flat-field-corrected tomograms affected by phase contrast are excluded from the analysis by applying morphological shrinking. Transmission values are taken from areas in the flat-field-corrected projection images where only solvent is present using the pixels with the 50% longest linear path lengths through the mother liquor. Next, Beer–Lambert’s law is applied on a pixel-by-pixel basis to calculate the absorption coefficients. The mother liquor absorption coefficient is then defined as the median of the resulting absorption coefficients. This value is used in the calculation of the absorption coefficients for the other materials (*e.g.* crystal or protein/detergent aggregate) according to their corresponding path lengths. A library of loop absorption coefficients based on tomography reconstructions of empty loops is available for the different loops used on the I23 beamline. The measured absorption coefficients are presented in Table 1 ▸. The composition and density of the protein/detergent aggregate are unknown, but its largest absorption coefficient of all materials is consistent with the flat-field-corrected projection image presented in Fig. 2 ▸(*c*).

### 2.4. Implementation details

A ray-tracing method is applied to compute the path lengths 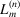 for each crystal voxel *n* of the reflection **h** in equation (3). For a crystal voxel *n*, it assumes an incoming and a diffracted X-ray beam originating from the voxel. These X-rays, after applying the rotational matrix of the goniometer 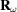 of the reflection **h**, will propagate through the 3D segmented model. The coordinates of each voxel, along with its corresponding material label, are recorded. Then, the path lengths 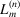 of material *m* can be determined by the distance between the coordinates of the boundaries of the materials. By combining the absorption coefficients of the corresponding materials, the absorption factor 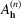 for the crystal voxel *n* can be determined [equation (3)]. Finally, the total absorption factor 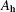 for the reflection **h** is calculated by summing 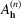 for all crystal voxels according to equation (2).

It is computationally intensive to rotate the overall 3D segmented model for each absorption factor calculation according to the rotational matrix of the goniometer 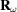 . Instead, *AnACor* rotates the vectors of the incoming and diffracted beams to calculate the path lengths by inverting the goniometer matrix. The tomography experiments are always performed at kappa/phi orientations κ = 0° and ϕ = 0°. To correct data from diffraction experiments with varying kappa/phi orientations, it is essential to transform the vectors of both the incoming and diffracted beams with the kappa/phi orientation matrices 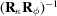 taken from the *DIALS* experiment model. Hence, the overall transformed vectors of these beams are in the form of 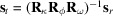 , where 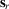 is either the vector of the incoming or that of the diffracted beam taken from the *DIALS* reflection data. The resulting directional vectors 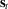 are used in the ray-tracing method. The incident beam is assumed to have a top-hat profile, so no additional beam profile correction is used. If the crystal is larger than the incident X-ray beam, a discriminator in the ray-tracing algorithm is used to determine whether a crystal voxel is inside the X-ray beam.

The absorption correction software *AnACor 1.0* is written in Python to facilitate future integration into *DIALS* (Winter *et al.*, 2018 ▸). In order to enhance computational efficiency, *NumPy 1.23.2* (Harris *et al.*, 2020 ▸) is used for data loading and preprocessing. *Numba 0.56.2* (Lam *et al.*, 2015 ▸) is used for JIT (just-in-time) compilation. A typical protein crystallography data set contains hundreds of thousands of reflections. There are typically millions of crystal voxels in a 3D model, and each path length calculation can involve determining thousands of voxels along the incident and diffracted X-ray paths. Consequently, calculating all absorption correction factors for samples in protein crystallography is computationally expensive. To mitigate this, a systematic sampling method with a sampling interval of 2000 is applied. This sampling approach relies on the sorted arrangement of the crystal voxels, which helps in identifying the subsections of the crystal where the path lengths (*L*_1_ and *L*_2_) are similar. Selecting every 2000th voxel from this sorted list ensures that sampling is consistently applied across the crystal. Therefore, it can capture the essential characteristics of the sample with far fewer data points, maintaining accuracy in equation (2) calculations while reducing computational load.

Parallel computing is used by the built-in *multiprocessing* package in Python, and the calculations of all the reflections are evenly distributed to each CPU core. After applying sampling and parallel computing, on a cluster node with 48 CPU cores, the computational time for the analytical absorption correction of one data set of OmpK36 and Cld is about 40 and 30 min, respectively, with total RAM usage of around 200 GB.

To evaluate the accuracy of the ray-tracing method with and without tomographic volume sampling, the absorption factor calculations were compared with previously published numerical solutions (Maslen, 2004 ▸). Three simulated shapes were considered: cubic, cylindrical and spherical, consisting of crystal material only. For consistency, a voxel size of 0.3 × 0.3 µm and the same sampling interval of 2000 were applied. Both approaches gave errors smaller than 0.5% for cubic and cylindrical shapes. The errors for the spherical shape were smaller than 0.75% with the exception of those at 90°. The results for a smaller voxel size of 0.1 × 0.1 µm indicate that the error is dominated by the pixel size rather than the sampling. More details can be found in the supporting information, section S1.

The codes and further explanations of the algorithm are available at https://github.com/yishunlu-222/AnACor_public.

### 2.5. Absorption correction strategies

Data scaling is performed by the *dials.scale* program in *DIALS* (Beilsten-Edmands *et al.*, 2020 ▸) using the following custom scaling model: 

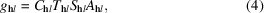

where 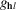 is the overall inverse scale factor that needs to be determined for the *l*th observation of symmetry-unique reflection **h**. The scale factors are determined by optimizing the scaling model parameters using a least-squares target function as previously described (Beilsten-Edmands *et al.*, 2020 ▸). 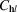 , 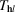 and 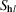 are, respectively, the scale term, the decay term and the spherical harmonics correction term of the default physical model. The absorption correction factors 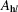 are precalculated by *AnACor* for each reflection 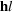 and not optimized during the scaling process.

The scale term 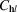 models intensity variations as a function of rotation, while the decay term 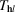 is a function of resolution and rotation. The spherical harmonics term 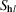 corrects the intensities with a model dependent on the incoming and scattered beam paths. The ‘absorption_level = high’ option in *dials.scale* (Winter *et al.*, 2022 ▸) was used for all approaches that included this term, which reduces the program’s restraints on 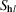 and uses six orders of spherical harmonics basis functions, to allow high and complex levels of absorption to be modelled. The ‘anomalous = False’ option in *dials.scale* was used, as the low multiplicity of individual data sets was found to lead to unstable error model refinement for some data sets when the option ‘anomalous = True’ was used.

To evaluate the analytical absorption correction by ray-tracing in *AnACor*, four approaches are compared:

(i) No absorption correction (labelled as NO) ( 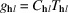 ).

(ii) Spherical harmonics correction (default in *dials.scale*, SH) ( 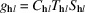 ).

(iii) Analytical absorption correction described in this work (AC) ( 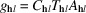 ).

(iv) Analytical absorption correction described in this work, combined with spherical harmonics correction (ACSH) ( 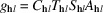 ).

The parameters for each part of the scaling model (except 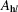 ) are jointly refined against the integrated intensities in each case and therefore will be different in each approach, *i.e.* 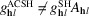 . The combination of the analytical absorption correction with spherical harmonics allows the effect of absorption to be corrected by an accurate analytical model, while still enabling the spherical harmonics model to correct for any residual effects.

## 3. Results

In crystallography, various metrics, such as *R* factors (Weiss & Hilgenfeld, 1997 ▸; Diederichs & Karplus, 1997 ▸; Weiss, 2001 ▸), correlation coefficients (Karplus & Diederichs, 2012 ▸) and signal-to-noise ratios, are used to evaluate data quality. Additionally, for long-wavelength crystallography peak heights in the phased anomalous difference Fourier maps are important quality indicators (Yang *et al.*, 2003 ▸). These metrics are used in combination with the success of experimental phasing by SAD to assess the three different absorption correction strategies and compare them with scaling without absorption correction.

Merging and refinement statistics (based on three data sets for OmpK36 and 22 for Cld) are presented in Table 2 ▸. As expected, for both samples, all four strategies result in similar resolution ranges, completeness and number of unique reflections. All three approaches to deal with absorption unsurprisingly lead to significant improvements in data quality over the data without correction.

**Figure 4:**
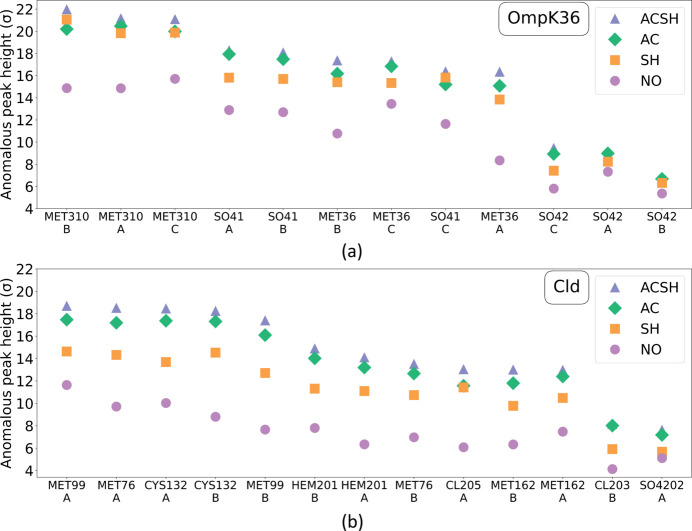
Peak heights (>5σ) in the anomalous difference Fourier maps of anomalous scatterers in OmpK36 (*a*) and Cld (*b*) plotted in descending order of peak heights in the ACSH data, generated by *Anode* (Thorn & Sheldrick, 2011 ▸). Raw data are presented in the supporting information, Tables S3 to S6 (OmpK36) and S7 to S10 (Cld).

For OmpK36, the analytical absorption correction (AC) gives equivalent merging *R* factors to spherical harmonics correction (SH), with an overall *R*_merge_ of 0.119 for both. Notably, the AC strategy leads to an increase in the mean *I*/σ(*I*), from 16.42 (SH) to 21.37 (AC), and a stronger anomalous signal, as measured by the anomalous slope (1.69 with AC, as opposed to 1.31 with SH). The anomalous slope (Evans, 2006 ▸) is the slope of the central region of a normal probability plot of anomalous differences: a slope greater than one indicates that the anomalous differences are larger than their uncertainties in aggregate. The combination of AC and SH corrections (ACSH) gives further improvements in the merging *R* factors, signal-to-noise ratio and anomalous signal, with the *R*_merge_ decreasing to 0.105, the mean *I*/σ(*I*) increasing to 24.92 and the anomalous slope increasing to 1.91. In Fig. 4 ▸(*a*), the anomalous peak heights from sulfur atoms for the three correction strategies are compared with no absorption correction for OmpK36. In total 12 sulfur atoms are found, from two methionine residues and two sulfates in the trimeric structure. A significant increase in peak heights is observed with all three absorption correction methods. AC generally gives better results than SH, with the exception of the heights of MET310 in chain B and SO4-1 in chain C, which are larger in the SH data. Overall, the ACSH strategy brings further improvements in peak heights, except for the weakest anomalous peak, SO4-2, where AC and ACSH perform similarly. Detailed information on the anomalous peaks of OmpK36 can be found in Tables S3–S6 in the supporting information. The refinement statistics for all strategies follow a similar trend to the merging statistics, with *R* factors being the lowest for ACSH. SAD phasing was performed as a further test of the efficacy of analytical absorption corrections. Phasing was attempted with one, two out of three and all three data sets available. The results, summarized in Table 3 ▸, show that the ACSH strategy outperforms the others in requiring only two data sets for successful phasing despite the overall completeness of 89.2% and multiplicity of 8.3. Both AC and SH need all three data sets (98.9% overall completeness, multiplicity of 11.0), while the NO strategy is unsuccessful. The numbers of correct residues automatically built into the experimental maps are identical between the three successful strategies, indicating that the quality of the maps is of similar standard and the lower data completeness used for the ACSH approach has no impact.

For Cld, the merging *R* factors, *I*/σ(*I*) and anomalous slopes are noticeably better for AC compared with SH. All merging statistics show further improvement for the combined ACSH correction. In contrast to OmpK36, where data quality indicators changed little between the SH and AC strategies, for Cld, the analytical absorption correction strategy (AC) gives substantially better data statistics compared with SH. For instance, in terms of the merging *R* factors, we observe a decrease of the *R*_merge_ from 0.163 with SH to 0.112 with AC and a further decrease to 0.095 with the ACSH treatment. There is also an increase in the overall mean *I*/σ(*I*) from 20.22 for SH to 44.73 for the ACSH strategy with the high-resolution shell *I*/σ(*I*) following this trend. The anomalous slope value increases from 1.36 with SH to 2.48 and 2.5 for AC and ACSH, respectively. This indicates an impressive improvement in the anomalous signal as a result of applying analytical absorption corrections.

The anomalous peak heights for the different absorption correction strategies for Cld are shown in Fig. 4 ▸(*b*). In addition to three methionines and one cysteine per polypeptide chain, each Cld monomer also binds an Fe-containing heme ligand and a Cl^−^ anion. A single SO_4_^2−^ anion could be identified for the dimer, bringing the total number of anomalous scatterers to 13. SH leads to higher anomalous peak heights compared with no absorption correction. In line with the improved merging statistics, the anomalous signal in AC and ACSH is stronger than that in SH. ACSH gives the highest anomalous peak heights overall. While for OmpK36 the improvements in peak heights given by the AC and ACSH strategies over SH are quite modest, for Cld the increase from SH to AC/ACSH is more substantial. For the largest peaks, MET99 and CYS132, we observe increases in peak heights from 14 to 17 and 18σ for AC and ACSH, respectively. Further details of anomalous peak heights for Cld may be found in the supporting information, Tables S7–S10. The experimental phasing results for this sample (presented in Table 3 ▸) show that the AC and ACSH strategies perform very similarly, with a successful phasing outcome requiring only two out of 22 data sets, with an overall completeness of 83.3% and overall multiplicity of 4.4. For the SH strategy, three data sets are needed, with a higher overall completeness of 94.7% and multiplicity of 5.8. These results follow the same pattern seen with the data quality indicators discussed above, where the AC strategy outperforms the SH approach. Experimental phasing is unsuccessful for the Cld data with no absorption corrections, even after merging all 22 data sets.

**Figure 5:**
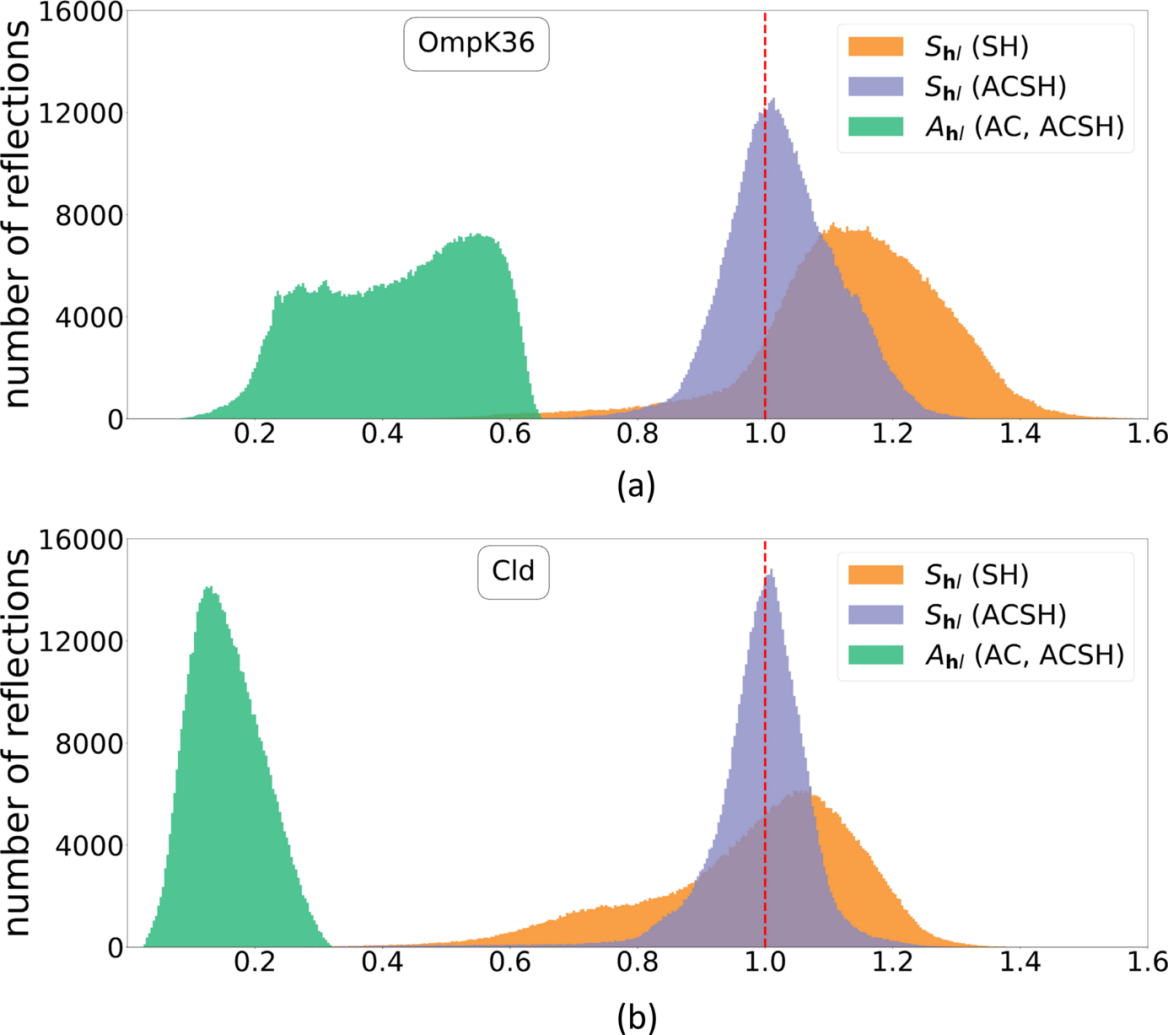
Histograms of absorption factors 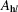 and spherical harmonics terms 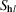 for OmpK36 (*a*) and Cld (*b*). 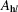 (green) as used in AC and ACSH strategies are on an absolute scale, whereas 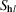 for SH (orange) and ACSH (purple) are on a relative scale.

To illustrate the extent of the AC and SH corrections, histograms of the per-reflection analytical absorption correction factors ( 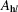 ) and spherical harmonics correction terms ( 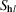 ) are presented in Fig. 5 ▸ for OmpK36 and Cld. For both data sets, when employing the SH correction strategy, the resulting spherical harmonics terms ( 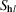 ) are distributed over a large range (0.5–1.5). When employing the ACSH strategy, the inclusion of the absorption correction factors ( 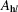 ) (shown on the right of Fig. 5 ▸) leads to unimodal 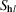 distributions over a narrower range (0.7–1.3) centred around 1. As the ‘no correction value’ for the SH model is 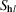 = 1.0, fitting the additional spherical harmonics terms in the ACSH strategy results in further improvement in the internal consistency compared with AC alone, allowing correction for additional systematic effects present in the data.

## 4. Discussion and conclusion

In this study we demonstrate the successful application of analytical absorption corrections based on 3D reconstructions from X-ray tomography implemented in *AnACor*. We describe the algorithm for calculating the path lengths from 3D models by a ray-tracing method. Two very long wavelength experiments from crystals of the proteins OmpK36 and Cld indicate that this approach substantially improves data quality and the success of experimental phasing compared with the standard scaling protocol based on spherical harmonics. Scaling without any absorption correction is presented as a control and unsurprisingly yields the poorest data quality statistics and anomalous peak heights, and for both samples experimental phasing is unsuccessful. This clearly indicates that data quality is severely affected by absorption effects, demonstrating the need for absorption corrections.

Data from OmpK36, which crystallizes in the monoclinic space group *C*2, were collected at a wavelength of λ = 3.54 Å. A clear trend is visible: the analytical absorption correction (AC) is better than the spherical harmonics correction (SH) and the combination of the two (ACSH) improves the data even further. While the overall improvements on statistics are small, the fact that the OmpK36 structure could be solved after ACSH correction using only 2/3 of the data needed for the AC and SH strategies clearly highlights the importance of such an improvement. For the Cld data (*P*1, λ = 4.13 Å) the same trend is observed. However, while the difference between AC and ACSH is small, they outperform the spherical harmonics correction. This is in particular reflected in the outcome from experimental phasing, where two data sets are sufficient for both AC and ACSH, while three data sets are needed to solve the structure from data corrected by SH. In general, the combined approach of ACSH gives the best results for both samples/wavelengths, as it can model additional systematic effects present in the experimental data.

X-ray absorption increases with the cube of the wavelength, so a change from λ = 1.0 Å to λ = 4.13 Å leads to a 70-fold increase in absorption coefficients. The analytical absorption correction compensates for this increase, reflected in the narrow unimodal distribution of the resulting spherical harmonics terms 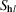 centred around 1.0 in the two ACSH cases. Both samples used in this study crystallize in either monoclinic (OmpK36) or triclinic (Cld) space groups. This in combination with the asymmetry of the cylindrical P12M detector, with an aspect ratio of 2:1, leads to a low overall data multiplicity of five for OmpK36 and only three in the case of Cld, as well as poor data completeness for a single 360° data set. In contrast to the spherical harmonics, the analytical absorption correction is not dependent on multiple observations, and hence is ideally suited for crystals in low-symmetry space groups or for radiation-sensitive crystals at long wavelengths.

*AnACor* is able to correct data in multiple crystal orientations and for cases where the beam is smaller than the sample. Future work will allow the use of experimentally determined beam profiles and increase the efficiency and speed of the software. Currently, the bottleneck is the manual segmentation step to create the 3D models. The increased phase contrast at long wavelengths and limitations with the current beamline hardware, in particular the sphere of confusion of the goniometer, lead to blurred boundaries in the tomographic reconstructions. The resulting inaccuracies in the segmented 3D model can affect both the path length and the absorption coefficient calculations. The next stage of this work is therefore to understand, quantify and reduce these errors impacting the 3D model. Analytical absorption corrections are beneficial not only for long-wavelength macromolecular crystallography but also for highly absorbing samples in chemical crystallography. In this work the segmented 3D model is obtained by X-ray tomography on beamline I23 at Diamond Light Source. However, *AnACor* can also be used for analytical absorption corrections for data from other sources, as long as a file with annotated voxels is provided and the relation between the coordinate systems of the 3D model and the diffraction experiment is known.

## Supplementary Material

Supporting information. DOI: 10.1107/S1600576724002243/yr5123sup1.pdf

PDB reference: OmpK36 GD NO, 8qur

PDB reference: OmpK36 GD SH, 8quq

PDB reference: OmpK36 GD AC, 8qvv

PDB reference: OmpK36 GD ACSH, 8qvs

PDB reference: chlorite dismutase NO, 8quv

PDB reference: chlorite dismutase SH, 8quu

PDB reference: chlorite dismutase AC, 8quz

PDB reference: chlorite dismutase ACSH, 8qvb

## Figures and Tables

**Table 1:** Linear absorption coefficients (µm^−1^) of different materials in OmpK36 (λ = 3.54 Å) and Cld (λ = 4.13 Å) samples

Sample	Crystal	Mother liquor	Loop	Protein/detergent aggregate
OmpK36	0.01053	0.01208	0.00931	0.0322
Cld	0.0160	0.01856	0.01724	N/A

**Table 2:** Merging and refinement statistics from OmpK36 and Cld Columns represent the four absorption correction methods: spherical harmonics correction (SH), analytical absorption correction (AC), analytical absorption correction combined with spherical harmonics correction (ACSH), no absorption correction (NO). Values in parentheses are for the outer resolution shell. Further refinement statistics can be found in the supporting information (Tables S1 and S2). For the calculation of the anomalous slope, the resolution range is restricted to resolutions below which the anomalous signal is significant in the ACSH processed data, which is 3.9 Å for OmpK36 and the full resolution range for Cld.

	NO	SH	AC	ACSH
OmpK36 (λ = 3.54 Å)				
*Merging statistics*				
Resolution range (Å)	107.4–2.34 (2.424–2.34)	107.4–2.34 (2.424–2.34)	107.4–2.34 (2.424–2.34)	107.4–2.34 (2.424–2.34)
Multiplicity	10.8 (5.5)	11.0 (5.5)	11.0 (5.5)	11.1 (5.5)
Completeness (%)	98.77 (91.67)	98.85 (92.15)	98.85 (92.12)	98.86 (92.15)
Mean *I*/σ(*I*)	11.99 (1.03)	16.42 (1.58)	21.37 (2.00)	24.92 (2.66)
*R*_merge_	0.139 (0.473)	0.119 (0.419)	0.119 (0.458)	0.105 (0.427)
*R*_meas_	0.146 (0.525)	0.125 (0.462)	0.125 (0.506)	0.110 (0.472)
*R*_pim_	0.043 (0.214)	0.035 (0.185)	0.035 (0.204)	0.031 (0.191)
CC_1/2_	0.996 (0.814)	0.997 (0.896)	0.997 (0.874)	0.998 (0.878)
CC*	0.999 (0.947)	0.999 (0.972)	0.999 (0.966)	0.999 (0.967)
Anomalous slope (*d* ≤3.9 Å)	1.13	1.31	1.69	1.91
Total reflections	654312 (31265)	668732 (31264)	668892 (31264)	672491 (31264)
Unique reflections	60652 (5606)	60652 (5634)	60652 (5633)	60652 (5634)
*Refinement statistics*				
Work set reflections	60585 (5605)	60631 (5634)	60630 (5632)	60639 (5634)
Free set reflections	3258 (328)	3260 (328)	3260 (328)	3260 (328)
*R*_work_	0.219 (0.390)	0.207 (0.338)	0.203 (0.332)	0.199 (0.294)
*R*_free_	0.255 (0.386)	0.244 (0.335)	0.240 (0.335)	0.235 (0.303)
PDB code	8qur	8quq	8qvv	8qvs
				
Cld (λ = 4.13 Å)				
*Merging statistics*				
Resolution range (Å)	46.67–2.7 (2.797–2.7)	46.67–2.7 (2.797–2.7)	46.67–2.7 (2.797–2.7)	46.67–2.7 (2.797–2.7)
Multiplicity	38.8 (23.5)	40.3 (23.5)	41.1 (23.5)	41.1 (23.5)
Completeness (%)	99.43 (97.97)	99.43 (97.97)	99.43 (97.97)	99.43 (97.97)
Mean *I*/σ(*I*)	16.51 (4.83)	20.22 (6.61)	37.43 (13.47)	44.73 (15.68)
*R*_merge_	0.205 (0.281)	0.163 (0.240)	0.112 (0.197)	0.095 (0.183)
*R*_meas_	0.208 (0.287)	0.165 (0.245)	0.113 (0.201)	0.096 (0.187)
*R*_pim_	0.033 (0.056)	0.025 (0.048)	0.017 (0.039)	0.014 (0.037)
CC_1/2_	0.997 (0.986)	0.997 (0.99)	0.999 (0.992)	0.999 (0.993)
CC*	0.999 (0.996)	0.999 (0.998)	1 (0.998)	1 (0.998)
Anomalous slope	1.28	1.36	2.48	2.50
Total reflections	531035 (31693)	551553 (31730)	562964 (31747)	563200 (31739)
Unique reflections	13696 (1351)	13696 (1351)	13696 (1351)	13696 (1351)
*Refinement statistics*				
Work set reflections	13696 (1351)	13696 (1351)	13696 (1351)	13696 (1351)
Free set reflections	686 (76)	686 (76)	686 (76)	686 (76)
*R*_work_	0.191 (0.240)	0.176 (0.223)	0.172 (0.210)	0.172 (0.209)
*R*_free_	0.234 (0.297)	0.223 (0.285)	0.218 (0.271)	0.218 (0.273)
PDB code	8quv	8quu	8quz	8qvb

**Table 3:** SAD phasing results for OmpK36 (top) and Cld (bottom): statistics from *Crank2* for all four absorption correction strategies While for some strategies only two data sets were needed for successful phasing, the statistics from using three data sets are presented for comparison.

Strategy	No. of data sets required for phasing	Completeness (overall/high-resolution bin)	Multiplicity (overall/high-resolution bin)	Refinement *R* factor/*R*_free_	No. of correct residues automatically built/total No. of residues
OmpK36					
NO	–	–	–	–	–
SH	3	98.8/88.1	11.0/3.9	0.235/0.280	1041/1041
AC	3	98.8/88.1	11.0/3.9	0.227/0.274	1041/1041
ACSH	2	89.2/71.9	8.3/3.3	0.228/0.280	1041/1041
ACSH	3	98.8/88.1	11.0/3.9	0.218/0.257	1041/1041
					
Cld					
NO	–	–	–	–	–
SH	3	94.7/82.2	5.8/2.5	0.259/0.336	354/376
AC	2	83.3/64.9	4.4/2.2	0.266/0.348	354/376
AC	3	94.7/82.2	5.9/2.5	0.260/0.320	362/376
ACSH	2	83.3/64.9	4.4/2.2	0.260/0.338	354/376
ACSH	3	94.7/82.2	5.9/2.5	0.259/0.302	362/376

## References

[bb1] Albrecht, G. (1939). *Rev. Sci. Instrum.* **10**, 221–222.

[bb2] Angel, R. J. (2004). *J. Appl. Cryst.* **37**, 486–492.

[bb3] Arndt, U. W. (1984). *J. Appl. Cryst.* **17**, 118–119.

[bb4] Beilsten-Edmands, J., Winter, G., Gildea, R., Parkhurst, J., Waterman, D. & Evans, G. (2020). *Acta Cryst.* D**76**, 385–399. 10.1107/S2059798320003198 PMC7137103 32254063

[bb5] Blessing, R. H. (1995). *Acta Cryst.* A**51**, 33–38. 10.1107/s0108767394005726 7702794

[bb6] Brockhauser, S., Di Michiel, M., McGeehan, J. E., McCarthy, A. A. & Ravelli, R. B. G. (2008). *J. Appl. Cryst.* **41**, 1057–1066.

[bb7] Bruker (2012). *APEX*. Bruker AXS Inc., Madison, Wisconsin, USA.

[bb8] Busing, W. R. & Levy, H. A. (1957). *Acta Cryst.* **10**, 180–182.

[bb9] Clark, R. C. & Reid, J. S. (1995). *Acta Cryst.* A**51**, 887–897.

[bb10] DeTitta, G. T. (1985). *J. Appl. Cryst.* **18**, 75–79.

[bb11] Diederichs, K. & Karplus, P. A. (1997). *Nat. Struct. Mol. Biol.* **4**, 269–275. 10.1038/nsb0497-269 9095194

[bb12] Duman, R., Orr, C. M., Mykhaylyk, V., El Omari, K., Pocock, R., Grama, V. & Wagner, A. (2021). *J. Vis. Exp.* **170**, e62364. 10.3791/62364 33970149

[bb13] El Omari, K., Duman, R., Mykhaylyk, V., Orr, C. M., Latimer-Smith, M., Winter, G., Grama, V., Qu, F., Bountra, K., Kwong, H. S., Romano, M., Reis, R. I., Vogeley, L., Vecchia, L., Owen, C. D., Wittmann, S., Renner, M., Senda, M., Matsugaki, N., Kawano, Y., Bowden, T. A., Moraes, I., Grimes, J. M., Mancini, E. J., Walsh, M. A., Guzzo, C. R., Owens, R. J., Jones, E. Y., Brown, D. G., Stuart, D. I., Beis, K. & Wagner, A. (2023). *Commun. Chem.* **6**, 219. 10.1038/s42004-023-01014-0 PMC10570326 37828292

[bb14] Evans, P. (2006). *Acta Cryst.* D**62**, 72–82. 10.1107/S0907444905036693 16369096

[bb15] Evans, P. R. & Murshudov, G. N. (2013). *Acta Cryst.* D**69**, 1204–1214. 10.1107/S0907444913000061 PMC3689523 23793146

[bb16] Gürsoy, D., De Carlo, F., Xiao, X. & Jacobsen, C. (2014). *J. Synchrotron Rad.* **21**, 1188–1193. 10.1107/S1600577514013939 PMC4181643 25178011

[bb17] Harris, C. R., Millman, K. J., van der Walt, S. J., Gommers, R., Virtanen, P., Cournapeau, D., Wieser, E., Taylor, J., Berg, S., Smith, N. J., Kern, R., Picus, M., Hoyer, S., van Kerkwijk, M. H., Brett, M., Haldane, A., del Río, J. F., Wiebe, M., Peterson, P., Gérard-Marchant, P., Sheppard, K., Reddy, T., Weckesser, W., Abbasi, H., Gohlke, C. & Oliphant, T. E. (2020). *Nature*, **585**, 357–362. 10.1038/s41586-020-2649-2 PMC7759461 32939066

[bb18] Howells, R. G. (1950). *Acta Cryst.* **3**, 366–369.

[bb19] Kabsch, W. (2010). *Acta Cryst.* D**66**, 125–132. 10.1107/S0907444909047337 PMC2815665 20124692

[bb20] Karplus, P. A. & Diederichs, K. (2012). *Science*, **336**, 1030–1033. 10.1126/science.1218231 PMC3457925 22628654

[bb21] Katayama, C., Sakabe, N. & Sakabe, K. (1972). *Acta Cryst.* A**28**, 293–295.

[bb22] Kazantsev, D., Duman, R., Wagner, A., Mykhaylyk, V., Wanelik, K., Basham, M. & Wadeson, N. (2021). *J. Synchrotron Rad.* **28**, 889–901. 10.1107/S1600577521003453 PMC8127374 33949996

[bb23] Kazantsev, D., Wadeson, N. & Basham, M. (2022). *SoftwareX*, **19**, 101157.

[bb24] Kopfmann, G. & Huber, R. (1968). *Acta Cryst.* A**24**, 348–351.

[bb25] Lam, S. K., Pitrou, A. & Seibert, S. (2015). *Proceedings of the Second Workshop on the LLVM Compiler Infrastructure in HPC*, pp. 1–6. Association for Computing Machinery.

[bb26] Leal, R. M. F., Teixeira, S. C. M., Rey, V., Forsyth, V. T. & Mitchell, E. P. (2008). *J. Appl. Cryst.* **41**, 729–737.

[bb27] Maslen, E. N. (2004). *International Tables for Crystallography*, 3rd ed., edited by E. Prince, Vol. C, ch. 6.3.3, pp. 600–608. Dordrecht: Kluwer.

[bb28] Merrifield, D. R., Ramachandran, V., Roberts, K. J., Armour, W., Axford, D., Basham, M., Connolley, T., Evans, G., McAuley, K. E., Owen, R. L. & Sandy, J. (2011). *Meas. Sci. Technol.* **22**, 115703.

[bb29] Meulenaer, J. de & Tompa, H. (1965). *Acta Cryst.* **19**, 1014–1018.

[bb30] Minor, W., Cymborowski, M., Otwinowski, Z. & Chruszcz, M. (2006). *Acta Cryst.* D**62**, 859–866. 10.1107/S0907444906019949 16855301

[bb31] Monaco, H. L. & Artioli, G. (2002). *Fundamentals of Crystallography*, 2nd ed., edited by H. Giacovazzo, ch. 5, pp. 376–388. Oxford University Press.

[bb32] Murshudov, G. N., Vagin, A. A. & Dodson, E. J. (1997). *Acta Cryst.* D**53**, 240–255. 10.1107/S0907444996012255 15299926

[bb33] North, A. C. T., Phillips, D. C. & Mathews, F. S. (1968). *Acta Cryst.* A**24**, 351–359.

[bb34] Schaffner, I., Mlynek, G., Flego, N., Pühringer, D., Libiseller-Egger, J., Coates, L., Hofbauer, S., Bellei, M., Furtmüller, P. G., Battistuzzi, G., Smulevich, G., Djinović-Carugo, K. & Obinger, C. (2017). *ACS Catal.* **7**, 7962–7976. 10.1021/acscatal.7b01749 PMC5678291 29142780

[bb35] Sheldrick, G. M. (1996). *SADABS.* University of Göttingen, Germany.

[bb36] Skubák, P. & Pannu, N. S. (2013). *Nat. Commun.* **4**, 2777. 10.1038/ncomms3777 PMC3868232 24231803

[bb37] Strutz, T. (2011). *IEEE/ACM Trans. Comput. Biol. Bioinf.* **8**, 797–807. 10.1109/TCBB.2010.67 20714026

[bb38] Thorn, A. & Sheldrick, G. M. (2011). *J. Appl. Cryst.* **44**, 1285–1287. 10.1107/S0021889811041768 PMC3246834 22477786

[bb39] Vo, N. T., Atwood, R. C. & Drakopoulos, M. (2018). *Opt. Express*, **26**, 28396–28412. 10.1364/OE.26.028396 30470012

[bb40] Wagner, A., Duman, R., Henderson, K. & Mykhaylyk, V. (2016). *Acta Cryst.* D**72**, 430–439. 10.1107/S2059798316001078 PMC4784674 26960130

[bb41] Walker, N. & Stuart, D. (1983). *Acta Cryst.* A**39**, 158–166.

[bb42] Warren, A. J., Armour, W., Axford, D., Basham, M., Connolley, T., Hall, D. R., Horrell, S., McAuley, K. E., Mykhaylyk, V., Wagner, A. & Evans, G. (2013). *Acta Cryst.* D**69**, 1252–1259. 10.1107/S0907444913011359 PMC3689528 23793151

[bb43] Weiss, M. S. (2001). *J. Appl. Cryst.* **34**, 130–135.

[bb44] Weiss, M. S. & Hilgenfeld, R. (1997). *J. Appl. Cryst.* **30**, 203–205.

[bb45] Winter, G., Beilsten–Edmands, J., Devenish, N., Gerstel, M., Gildea, R. J., McDonagh, D., Pascal, E., Waterman, D. G., Williams, B. H. & Evans, G. (2022). *Protein Sci.* **31**, 232–250. 10.1002/pro.4224 PMC8740827 34747533

[bb46] Winter, G., Waterman, D. G., Parkhurst, J. M., Brewster, A. S., Gildea, R. J., Gerstel, M., Fuentes-Montero, L., Vollmar, M., Michels-Clark, T., Young, I. D., Sauter, N. K. & Evans, G. (2018). *Acta Cryst.* D**74**, 85–97. 10.1107/S2059798317017235 PMC5947772 29533234

[bb47] Wong, J. L., Romano, M., Kerry, L. E., Kwong, H.-S., Low, W.-W., Brett, S. J., Clements, A., Beis, K. & Frankel, G. (2019). *Nat. Commun.* **10**, 1–10. 10.1038/s41467-019-11756-y PMC6718652 31477712

[bb48] Yang, C., Pflugrath, J. W., Courville, D. A., Stence, C. N. & Ferrara, J. D. (2003). *Acta Cryst.* D**59**, 1943–1957. 10.1107/s0907444903018547 14573949

[bb49] Zeldin, O. B., Gerstel, M. & Garman, E. F. (2013). *J. Appl. Cryst.* **46**, 1225–1230.

