# Supplementary material for: Ray-tracing analytical absorption correction for X-ray crystallography based on tomographic reconstructions
Source: J Appl Crystallogr. 2024 Apr 15;57(Pt 3):649–58. doi: 10.1107/S1600576724002243 (PMC11151674; doi:10.1107/S1600576724002243)
Supplement: Supplementary file 1 [file j-57-00649-sup1.pdf]

JOURNAL OF APPLIED CRYSTALLOGRAPHY

Volume 57 (2024)

Supporting information for article:

Ray-tracing analytical absorption correction for X-ray crystallography based on tomographic reconstructions

Yishun Lu, Ramona Duman, James Beilsten-Edmands, Graeme Winter, Mark Basham, Gwyndaf Evans, Jos J.A.G. Kamps, Allen M. Orville, Hok-Sau Kwong, Konstantinos Beis, Wesley Armour and Armin Wagner

Supplementary Materials for

Ray-tracing analytical absorption correction by X-ray tomographic reconstruction

1 Proof of correctness

This section describes the proof of correctness of the ray-tracing method by comparing the results of the ray-tracing method used in this study with the numerical solution previously published in the International Tables [1]. Three regular hypothetical crystal volumes, cubic, cylindrical, and spherical, are simulated and presented in Fig. S1 (a) - (c).

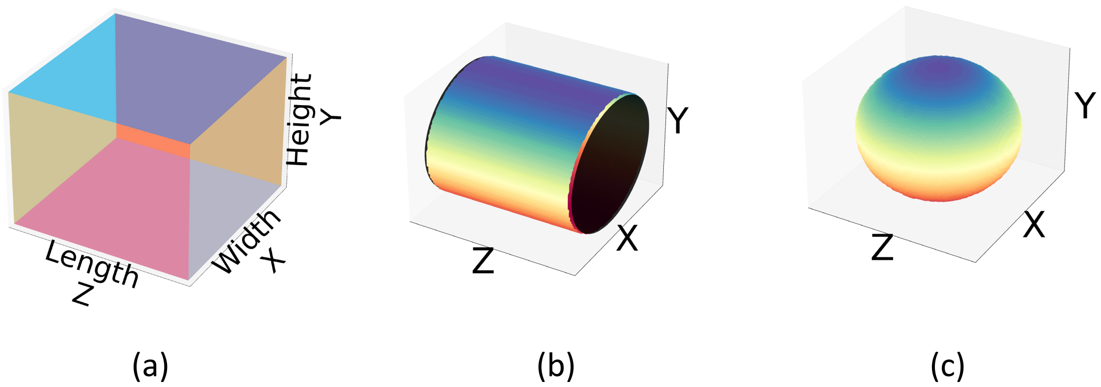

Figure S1: 3D visualization of a cube (a), cylinder (b), and sphere (c).

The calculations use voxel sizes of 0.3 μm^3 and 0.1 μm^3, where 0.3 μm^3 is the same as for the tomography reconstructions in this paper. In the simulation, a single material with an absorption coefficient of 0.01 μm^{-1}, close to the absorption coefficient of the OmpK36 crystal, measured at 3.53 Å, is assumed. For the cube, the resultant integrals can be solved analytically while for the cylinder and sphere, the numerical solutions are taken from the International Tables [1]. The incident X-rays are simulated as going along the X-axis, and the diffraction angles are the angles between the X-axis and the diffracted X-rays, as described in the International Tables. Three different dimensions, similar to those of the crystals in this study, are used for each object.

- Cube:
  - (A): Length=100 µm, Width=100 µm, Height=50 µm
  - (B): Length=100 µm, Width=100 µm, Height=100 µm
  - (C): Length=100 µm, Width=100 µm, Height=150 µm
- Cylinder:
  - (A): Length=50 µm, Radius=10 µm
  - (B): Length=50 µm, Radius=50 µm
  - (C): Length=50 µm, Radius=100 µm

- Sphere:
  - (A): Radius=10 µm
  - (B): Radius=50 µm
  - (C): Radius=100 µm

Percentage errors between the values from numerical solutions and those from the ray-tracing method presented in this study are plotted in Fig. S2, with both no sampling (a-c) and sampling method employed here (d-f). It can be observed that without sampling, the general percentage errors for all three volumes are smaller than 0.5%. After applying the sampling method, the overall percentage errors are still around 0.5%, except for some diffraction angles for the sphere. To evaluate the effect of the resolution of the 3D model, a smaller voxel size of 0.1 μm^3 is used (orange symbols in Fig. S2). To maintain the same number of crystal voxels that are involved in the calculation, the sampling ratio is decreased by a factor of 3^3 (sampling every 54000th coordinate). As voxel sizes decrease, the percentage errors also become smaller, showing that the effects of sampling are less significant than the impacts resulting from the voxel size.

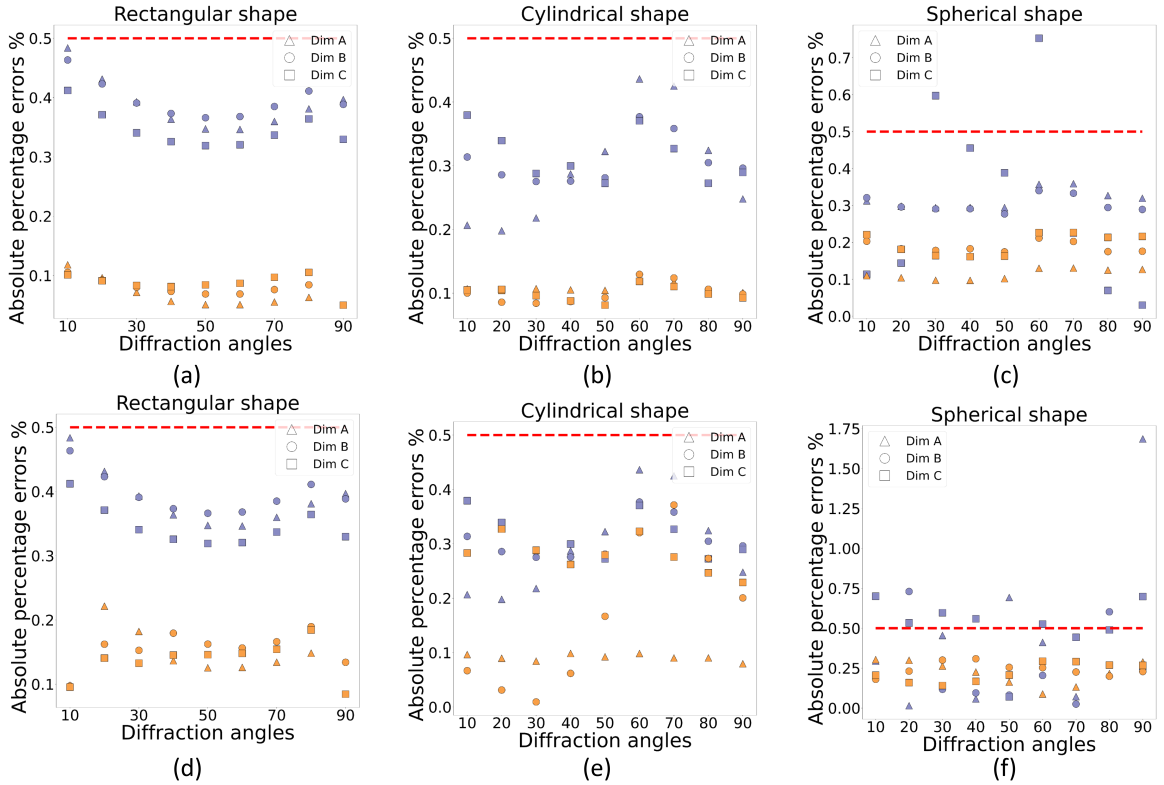

Figure S2: The absolute percentage difference of three dimensions with different voxel sizes (Purple: 0.3 μm^3; Orange: 0.1 μm^3) between the values in the International tables [1] and the results in our work. (a)-(c): no sampling, (d)-(f): with tomographic volume sampling. The top row and the bottom row are for the differences without and with tomographic volume sampling, respectively. The absolute percentage error is calculated by the $\frac{|table - raytracing|}{table} \times 100$

2 Crystallisation of Cld

Large Cld crystals (200 – 600µm, diamond-shaped morphology) were obtained using sitting drop vapour diffusion (24 well plate, CrysChem 24-well, Hampton Research) at 25 °C. Cld protein (3µL, 12mg/mL), in 10mM HEPES buffer pH 7.4, was mixed in a 1:1 ratio with crystallization solution (150 mM MgSO4,100 mM MES pH 6.5 and 20 w/v% PEG 3350) and equilibrated against crystallization solution (350µL). Crystals were obtained over several days (4-7) and allowed to mature. Cld crystals (5 × 200 – 600µm) selected for the preparation of seed stocks were transferred into crystallization solution (100µL; Eppendorf tube 1.5mL) and crushed using glass seed beads (4 ×, Hampton Research) over multiple (10 ×) vortex agitation (30s) and cooling on ice (4 °C, 30s) cycles. Cld crystals (50 – 200µm) were obtained using sitting drop vapour diffusion at 25 °C. Cld protein (3µL, 12mg/mL) in 10mM HEPES buffer, pH 7.4, was mixed in a 1:1 ratio with crystallization solution (150mM MgSO4, 100mM MES, pH 6.5 and 20 w/v%PEG 3350) and dilute seed stock (0.5µL) and equilibrated against crystallization solution (350µL). Cld crystals were matured over 24 h. Cryo-protection was performed by by first transferring into glycerol solution (10 v/v% glycerol , 90 v/v% crystallization solution, 30s), then into NaCl solution (30 v/v% /150mM final NaCl, 20 v/v% glycerol and 50 v/v% crystallization solution), before plunge-freezing into liquid nitrogen.

3 REFINEMENT DETAILS

Table S1: Detailed refinement tables of OmpK36 with different absorption correction strategies.

	No	SH	AC	ACSH
Wavelength	3.54	3.54	3.54	3.54
Resolution range	107.4 - 2.34 (2.424 - 2.34)	107.4 - 2.34 (2.424 - 2.34)	107.4 - 2.34 (2.424 - 2.34)	107.4 - 2.34 (2.424 - 2.34)
Space group	*C* 1 2 1	*C* 1 2 1	*C* 1 2 1	*C* 1 2 1
Unit cell	232.078 74.7004 91.3496 90 112.209 90	232.078 74.7004 91.3496 90 112.209 90	232.078 74.7004 91.3496 90 112.209 90	232.078 74.7004 91.3496 90 112.209 90
Total reflections	654312 (31265)	668732 (31264)	668892 (31264)	672491 (31264)
Unique reflections	60652 (5606)	60652 (5634)	60652 (5633)	60652 (5634)
Multiplicity	10.8 (5.5)	11.0 (5.5)	11.0 (5.5)	11.1 (5.5)
Completeness (%)	98.77 (91.67)	98.85 (92.15)	98.85 (92.12)	98.86 (92.15)
Mean I/sigma(I)	11.99 (1.03)	16.42 (1.58)	21.37 (2.00)	24.92 (2.66)
Wilson B-factor	46.83	42.25	42.62	40.13
R-merge	0.1393 (0.4732)	0.1192 (0.4188)	0.1191 (0.4584)	0.1054 (0.4266)
R-meas	0.1461 (0.5249)	0.1245 (0.4624)	0.1245 (0.5064)	0.1101 (0.4719)
R-pim	0.04265 (0.2137)	0.03485 (0.1849)	0.03517 (0.2036)	0.03071 (0.1907)
CC1/2	0.996 (0.814)	0.997 (0.896)	0.997 (0.874)	0.998 (0.878)
CC*	0.999 (0.947)	0.999 (0.972)	0.999 (0.966)	0.999 (0.967)
Reflections used in refinement	60585 (5605)	60631 (5634)	60630 (5632)	60639 (5634)
Reflections used for R-free	3258 (328)	3260 (328)	3260 (328)	3260 (328)
R-work	0.2189 (0.3897)	0.2068 (0.3383)	0.2032 (0.3318)	0.1985 (0.2944)
R-free	0.2553 (0.3861)	0.2443 (0.3352)	0.2403 (0.3354)	0.2352 (0.3028)
CC(work)	0.926 (0.554)	0.916 (0.686)	0.930 (0.625)	0.932 (0.705)
CC(free)	0.906 (0.628)	0.861 (0.695)	0.868 (0.648)	0.870 (0.695)
Number of non-hydrogen atoms	8327	8327	8327	8327
macromolecules	8099	8099	8099	8099
ligands	30	30	30	30
solvent	198	198	198	198
Protein residues	1038	1038	1038	1038
**Nucleic acid bases**				
RMS(bonds)	0.008	0.008	0.008	0.009
RMS(angles)	1.59	1.6	1.57	1.59
Ramachandran favored (%)	94.47	94.76	94.27	94.37
Ramachandran allowed (%)	5.15	4.85	5.34	5.24
Ramachandran outliers (%)	0.39	0.39	0.39	0.39
Rotamer outliers (%)	1.09	0.97	1.33	1.21
Clashscore	4.69	3.66	3.6	3.34
Average B-factor	47.64	42.93	43.02	41.23
macromolecules	47.58	42.88	42.97	41.16
ligands	79.01	70.35	70.42	68.6
solvent	45.03	40.94	41.14	39.98
PDB code	8C2X	8C2W	8C2V	8C2U

Table S2: Detailed refinement tables of Cld with different absorption correction strategies.

	No	SH	AC	ACSH
Wavelength	4.132	4.132	4.132	4.132
Resolution range	46.67 - 2.7 (2.797 - 2.7)	46.67 - 2.7 (2.797 - 2.7)	46.67 - 2.7 (2.797 - 2.7)	46.67 - 2.7 (2.797 - 2.7)
Space group	*P*1	*P*1	*P*1	*P*1
Unit cell	51.5567 52.755 54.9524 107.215 99.0256 109.305	51.5567 52.755 54.9524 107.215 99.0256 109.305	51.5567 52.755 54.9524 107.215 99.0256 109.305	51.5567 52.755 54.9524 107.215 99.0256 109.305
Total reflections	531035 (31693)	551553 (31730)	562964 (31747)	563200 (31739)
Unique reflections	13696 (1351)	13696 (1351)	13696 (1351)	13696 (1351)
Multiplicity	38.8 (23.5)	40.3 (23.5)	41.1 (23.5)	41.1 (23.5)
Completeness (%)	99.43 (97.97)	99.43 (97.97)	99.43 (97.97)	99.43 (97.97)
Mean I/sigma(I)	16.51 (4.83)	20.22 (6.61)	37.43 (13.47)	44.73 (15.68)
Wilson B-factor	30.56	30.75	31.16	30.98
R-merge	0.2051 (0.2809)	0.1628 (0.2398)	0.112 (0.1969)	0.09457 (0.1828)
R-meas	0.2078 (0.2869)	0.1647 (0.2449)	0.1133 (0.201)	0.09566 (0.1868)
R-pim	0.03265 (0.05648)	0.02486 (0.04813)	0.01669 (0.03934)	0.01412 (0.03688)
CC1/2	0.997 (0.986)	0.997 (0.99)	0.999 (0.992)	0.999 (0.993)
CC*	0.999 (0.996)	0.999 (0.998)	1 (0.998)	1 (0.998)
Reflections used in refinement	13696 (1351)	13696 (1351)	13696 (1351)	13696 (1351)
Reflections used for R-free	686 (76)	686 (76)	686 (76)	686 (76)
R-work	0.1909 (0.2402)	0.1756 (0.2229)	0.1717 (0.2100)	0.1716 (0.2090)
R-free	0.2335 (0.2969)	0.2226 (0.2848)	0.2177 (0.2712)	0.2184 (0.2729)
CC(work)	0.889 (0.717)	0.912 (0.744)	0.916 (0.737)	0.916 (0.744)
CC(free)	0.875 (0.697)	0.907 (0.712)	0.910 (0.720)	0.910 (0.722)
Number of non-hydrogen atoms	3179	3179	3179	3179
macromolecules	3016	3016	3016	3016
ligands	111	111	111	111
solvent	52	52	52	52
Protein residues	362	362	362	362
**Nucleic acid bases**				
RMS(bonds)	0.011	0.011	0.011	0.011
RMS(angles)	1.57	1.58	1.56	1.56
Ramachandran favored (%)	94.97	96.09	96.37	96.37
Ramachandran allowed (%)	5.03	3.91	3.63	3.63
Ramachandran outliers (%)	0	0	0	0
Rotamer outliers (%)	1.6	1.6	1.6	1.6
Clashscore	3.39	3.22	3.39	3.55
Average B-factor	29.15	29.63	30.1	29.96
macromolecules	29.39	29.88	30.35	30.22
ligands	27.61	27.49	27.68	27.39
solvent	18.5	19.65	20.61	20.43
PDB code	8C2C	8C2B	8C2A	8C1O

4 ANOMALOUS PEAK HEIGHTS FOR OmpK36

Table S3: Anomalous peak heights ($> 5\sigma$) for OmpK36 with no absorption correction (NO).

Atom	Residue	Chain	Peak heights
S	MET310	C	15.71
S	MET310	B	14.87
S	MET310	A	14.86
S	MET36	C	13.45
S	SO41	A	12.89
S	SO41	B	12.7
S	SO41	C	11.64
S	MET36	B	10.77
S	MET36	A	8.35
S	SO42	A	7.32
S	SO42	C	5.8
S	SO42	B	5.36

Table S4: Anomalous peak heights ($> 5\sigma$) for OmpK36 with spherical harmonics absorption correction (SH).

Atom	Residue	Chain	Peak heights
S	MET310	B	21.05
S	MET310	C	19.88
S	MET310	A	19.83
S	SO41	C	15.82
S	SO41	A	15.81
S	SO41	B	15.7
S	MET36	B	15.4
S	MET36	C	15.33
S	MET36	A	13.85
S	SO42	A	8.23
S	SO42	C	7.42
S	SO42	B	6.32

Table S5: Anomalous peak heights ($> 5\sigma$) for OmpK36 with analytical absorption correction (AC).

Atom	Residue	Chain	Peak heights
S	MET310	A	20.47
S	MET310	B	20.2
S	MET310	C	19.99
S	SO41	A	17.94
S	SO41	B	17.48
S	MET36	C	16.84
S	MET36	B	16.18
S	SO41	C	15.2
S	MET36	A	15.08
S	SO42	A	8.98
S	SO42	C	8.93
S	SO42	B	6.68

Table S6: Anomalous peak heights ($> 5\sigma$) for OmpK36 with analytical + spherical harmonics absorption correction (ACSH).

Atom	Residue	Chain	Peak heights
S	MET310	B	21.97
S	MET310	A	21.16
S	MET310	C	21.09
S	SO41	A	18.28
S	SO41	B	18.08
S	MET36	B	17.37
S	MET36	C	17.28
S	SO41	C	16.37
S	MET36	A	16.34
S	SO42	C	9.46
S	SO42	A	8.81
S	SO42	B	6.82

5 ANOMALOUS PEAK HEIGHTS FOR Cld

Table S7: Anomalous peak heights ($> 5\sigma$) for Cld with no absorption correction (NO).

Atom	Residue	Chain	Peak heights
S	MET99	A	11.64
S	CYS132	A	10.03
S	MET76	A	9.71
S	CYS132	B	8.8
FE	HEM201	B	7.8
S	MET99	B	7.66
S	MET162	A	7.47
S	MET76	B	6.97
FE	HEM201	A	6.34
S	MET162	B	6.33
CL	CL205	A	6.08
S	SO4202	A	5.11

Table S8: Anomalous peak heights ($> 5\sigma$) for Cld with spherical harmonics absorption correction (SH).

Atom	Residue	Chain	Peak heights
S	MET99	A	14.63
S	CYS132	B	14.52
S	MET76	A	14.32
S	CYS132	A	13.68
S	MET99	B	12.71
CL	CL205	A	11.42
FE	HEM201	B	11.31
FE	HEM201	A	11.09
S	MET76	B	10.74
S	MET162	A	10.48
S	MET162	B	9.78
CL	CL203	B	5.91
S	SO4202	A	5.68

Table S9: Anomalous peak heights ($> 5\sigma$) for Cld with analytical absorption correction (AC).

Atom	Residue	Chain	Peak heights
S	MET99	A	17.47
S	CYS132	A	17.37
S	CYS132	B	17.31
S	MET76	A	17.19
S	MET99	B	16.09
FE	HEM201	B	14.02
FE	HEM201	A	13.2
S	MET76	B	12.66
S	MET162	A	12.4
S	MET162	B	11.8
CL	CL205	A	11.57
CL	CL203	B	8.01
S	SO4202	A	7.18

Table S10: Anomalous peak heights ($> 5\sigma$) for Cld with analytical + spherical harmonics absorption correction (ACSH).

Atom	Residue	Chain	Peak heights
S	MET99	A	18.7
S	MET76	A	18.52
S	CYS132	A	18.46
S	CYS132	B	18.24
S	MET99	B	17.4
FE	HEM201	B	14.89
FE	HEM201	A	14.1
S	MET76	B	13.51
CL	CL205	A	13.04
S	MET162	B	13.01
S	MET162	A	12.98
CL	CL203	B	8.14
S	SO4202	A	7.63

References

[1] Maslen, E. N. (2006). *International Tables for Crystallography*, volume C, chapter 6.3.3, Absorption corrections, pages 600–608. 3rd edition.
